# Supplementary material for: Single Cell Transcriptomic and Chromatin Profiles Suggest Layer Vb Is the Only Layer With Shared Excitatory Cell Types in the Medial and Lateral Entorhinal Cortex
Source: Front Neural Circuits. 2022 Jan 26;15:806154. doi: 10.3389/fncir.2021.806154 (PMC8826650; doi:10.3389/fncir.2021.806154)
Supplement: Supplementary file 14 [file Data_Sheet_1.ZIP › Exc_vs_Inh_Motifs/homerResults/motif3.similar.html]

motif3

## Information for motif3

C
A
T
G
A
G
C
T
T
C
A
G
A
C
T
G
A
T
C
G
G
A
T
C
C
T
A
G
C
A
T
G
  
Reverse Opposite:  

G
T
A
C
G
A
T
C
C
T
A
G
A
T
G
C
A
G
T
C
A
G
T
C
C
T
G
A
G
T
A
C
  

|  |  |
| --- | --- |
| p-value: | 1e-903 |
| log p-value: | -2.080e+03 |
| Information Content per bp: | 1.844 |
| Number of Target Sequences with motif | 14053.0 |
| Percentage of Target Sequences with motif | 33.31% |
| Number of Background Sequences with motif | 2166.3 |
| Percentage of Background Sequences with motif | 19.93% |
| Average Position of motif in Targets | 317.8 +/- 242.4bp |
| Average Position of motif in Background | 333.7 +/- 245.3bp |
| Strand Bias (log2 ratio + to - strand density) | -0.0 |
| Multiplicity (# of sites on avg that occur together) | 1.33 |
| Motif File: | file (matrix) reverse opposite |

### Similar de novo motifs found

|  |  |  |  |  |  |  |  |
| --- | --- | --- | --- | --- | --- | --- | --- |
| Rank | Match Score | Redundant Motif | P-value | log P-value | % of Targets | % of Background | Motif file |
| 1 | 0.927 | T C A G G A C T A C T G A C G T C T A G A C T G A C T G G C T A C A T G C A T G | 1e-840 | -1936.265557 | 24.49% | 13.24% | motif file (matrix) |
| 2 | 0.757 | G T C A A G T C A C T G G T A C A T G C G A C T G T C A A G T C | 1e-376 | -866.051624 | 16.74% | 10.14% | motif file (matrix) |
| 3 | 0.602 | A C G T A C T G A G T C A C T G A C G T A C T G A C T G A C T G C G T A C G T A A C T G A C G T | 1e-183 | -421.507554 | 34.24% | 27.81% | motif file (matrix) |
| 4 | 0.620 | A G T C A G T C A C T G A C G T A C T G A C G T A C T G A C T G A C T G C G T A A C T G A C G T | 1e-172 | -396.857650 | 31.27% | 25.21% | motif file (matrix) |
